# Inter and Intra-Rater Reliability of Measuring Photometric Craniovertebral Angle Using a Cloud-Based Video Communication Platform

**DOI:** 10.5195/ijt.2021.6346

**Published:** 2021-06-22

**Authors:** Rylan Cote, Cassandra Vietas, Megan Kolakowski, Kayla Lombardo, Jacob Prete, Amit Dashottar

**Affiliations:** 1 Department of Physical Therapy, College of Natural, Behavioral, and Health Sciences, Simmons University, Boston, Massachusetts, Usa

**Keywords:** Craniovertebral angle, Forward head posture, Physical therapy, Posture, Telehealth

## Abstract

**Objective:**

Due to social distancing guidelines during the Coronavirus (COVID-19) pandemic, most providers and patients have wanted to avoid close contact. This makes physical therapy (PT) assessments difficult because of the lack of empirical evidence about the reliability of various clinical measurements performed in a virtual environment. One such procedure is the photometric measurement of craniovertebral (CV) angle. Craniovertebral angle measurement is usually performed in an outpatient setting and is defined as the acute angle formed between a straight line connecting the spinous process of C7 to the tragus of the ear, and the horizontal line passing through the spinous process of the C7. Although the photometric measurement of CV angles is considered both valid and reliable in the clinics, no empirical evidence exists about the CV angle measurement reliability when performed in a virtual environment. Thus, the purpose of this study was to assess the inter- and intra-rater reliability of photometric CV angle measurement using a cloud-based video communication platform. *Number of Subjects:* 66 subjects (57 females).

**Methods:**

All measurements were performed by two final year PT students who had completed the musculoskeletal part of the curriculum and were blinded to each other's measurements. Each subject was photographed in two postures over a HIPAA-compliant video-based telehealth platform: (1) normal/relaxed posture and (2) ideal posture (posture the subject considered good). Student researcher 1 measured the CV angle in both the relaxed posture and ideal posture, while student researcher 2 measured the CV angle only in the relaxed posture. Each subject's CV angle measurement was performed three times on three separate days and the means were used for further analysis. The shape of the CV angle frequency distribution was assessed using kurtosis and skewness values. Rater reliability was assessed using intraclass correlation coefficients (ICC), and interpreted based on the guidelines provided by Portney and Watkins (2009).

**Results:**

The CV angles were normally distributed in both relaxed and ideal postures. The mean and standard deviation (SD) of relaxed posture was 50.7o ± 6.3o with kurtosis and skewness of 0.67 and −0.74 respectively. The mean and SD of ideal posture was 55.5o ± 5.4o, with kurtosis and skewness of 0.1 and −0.54 respectively. The ICC for inter-rater reliability in the relaxed posture was 0.88 and the ICC for intra-rater reliability for relaxed posture was 0.91.

**Conclusions:**

Craniovertebral angles were normally distributed in the sample. An acceptable level of inter- and intra-rater reliability can be attained when measuring CV angle using a cloud-based video communication platform.

Forward head posture (FHP) is described as the malalignment of the cervical spine due to increased flexion of the lower cervical spine and extension of the upper cervical spine in the sagittal plane (Garrett et al., 1993; White et al., 2018). Forward head posture is associated with neck and shoulder pain but may also be present in asymptomatic individuals (Mahmoud et al., 2019; Nejati et al., 2015; Szikszay et al., 2019). Biomechanically, FHP may lead to alteration of the typical anatomical relationship between the head and the cervical spine that in turn may lead to changes in the length tension relationship of the cervical musculature (Bergqvist et al., 1995; Khayatzadeh et al., 2017; White et al., 2018). FHP is assessed as a part of postural examination in physical therapy clinics and is commonly corrected in symptomatic and asymptomatic individuals (Garrett et al., 1993; Silva et al., 2009; White et al., 2018).

There are several ways of assessing FHP including but not restricted to visual assessment with or without reference to a vertical plumb line; use of a cervical range of motion (CROM) device; and by quantifying the craniovertebral angle (CV angle) (Cureton, 1941; Garrett et al., 1993; C. Lee et al., 2017; K.-J. Lee et al., 2015; Salahzadeh et al., 2014; Singla et al., 2017; Szikszay et al., 2019; van Niekerk et al., 2008; White et al., 2018). Craniovertebral angle is described as the acute angle formed between a horizontal line passing through the spinous process of the seventh cervical vertebra (C7) and the line connecting the midpoint of the tragus to the spinous process of C7 (Figure 1) (Cureton, 1941; Garrett et al., 1993; Raine & Twomey, 1997; Singla et al., 2017; White et al., 2018). Forward head posture and CV angle magnitude are inversely related, (i.e., as the CV angle decreases, the severity of FHP increases). Craniovertebral angle is the most widely used measurement to assess FHP. Not only it is both valid and reliable; but also it is objective and does not require any specialized equipment such as a CROM device (C. Lee et al., 2017; Nejati et al., 2015; Raine & Twomey, 1997; Salahzadeh et al., 2014; Singla et al., 2017; van Niekerk et al., 2008).

**Figure 1:**
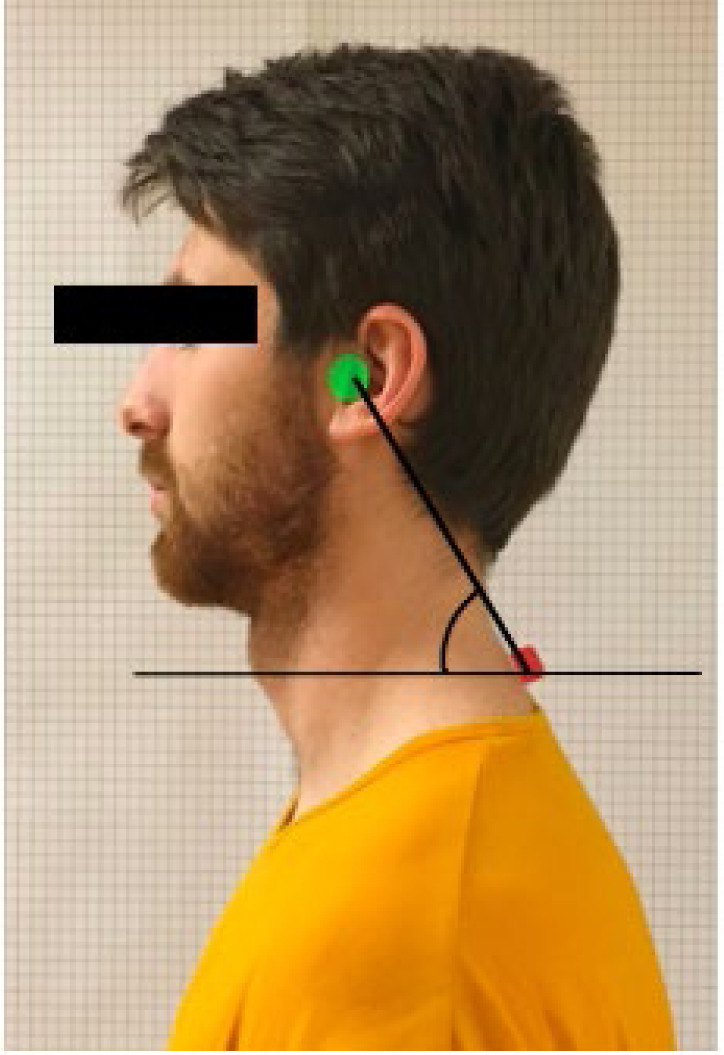
Craniovertebral Angle Landmarks

Even though CV angle is a valid measurement in clinical settings, the current circumstances present a unique problem. With the increased utilization of telehealth due to social distancing guidelines, physical therapists (PTs) are delivering care through telehealth (Exum et al., 2020; A. C. Lee, 2020). Increased use of telehealth means that PTs are now required to assess and treat patients using some type of video conferencing application such as a HIPAA-compliant video-based telehealth platform. It is not yet known whether the psychometric properties of CV angle measurement, validated under in-person clinical settings, translate to a virtual environment. We believe that specific PT clinical measurements should be validated in a telehealth setting before being applied in such settings.

The purpose of this study was to test the rater reliability of CV angle measurement using a video conferencing platform. Our hypothesis was that the measurement of CV angle over a video conferencing platform would have acceptable levels of rater reliability (Portney & Watkins, 2009).

## METHODS

The Simmons University IRB approved the study.

## SUBJECT RECRUITMENT

Sixty-six subjects who met the inclusion and exclusion criteria were recruited, and informed consent was obtained (Table 1.1). The inclusion criteria consisted of licensed clinical physical therapists and Doctor of Physical Therapy (DPT) students ages 18 and older. Subjects were recruited online and through word-of-mouth. There were no explicit exclusion criteria for participation in the study. Consent was obtained electronically via secure signature software and subjects who consented were sent an email with detailed study information, an intake form, and additional instructions to complete the video conference call. Following this, a HIPAA-compliant video-based telehealth appointment with the subject was scheduled. Prior to their scheduled meeting time, subjects filled out demographic data using an editable electronic file created by the researchers. Telehealth communications through some telehealth platforms can be secure and encrypted (Zoom Encryption, 2020).

**Table 1.1:** Demographic Data

Demographics	Mean	Standard Deviation	Range
Age	26.2	6.1	21 – 55
Height (inches)	65.7	3.0	61 – 74
Weight (lbs.)	143.3	29.2	101 – 240
Total Number of Subjects (n) = 66			

## DATA COLLECTION

Data collection began after the consent was obtained. The subjects were called at the scheduled time and two photographs showing the subject's head, neck and shoulder region were taken. Briefly, the data collection session began with an explanation of the procedure and taking photographs of the subject in both the relaxed and the ideal posture.

### SUBJECT POSITIONING

Each subject stood in front of a plain wall, feet hip-width apart, oriented in the sagittal plane with respect to the camera embedded in the subject's computer or phone. Researchers directed subjects to adjust the height of the camera as necessary to ensure the subject was centered in the frame and his/her shoulder, top of the head, and tragus were visible. The researcher used the computer's screen grab function to obtain the photographs.

### PROCEDURE

Subjects were instructed on palpating the spinous process of C7. Once palpated, the subject marked the location with a piece of colored tape and re-palpated to ensure the tape was placed correctly. For the photograph, the tragus of the ear was exposed, and the colored tape was visible (Figure 1). The following instructions were given to subjects prior to capturing the photograph: “March in place for a few steps then assume your best posture.” Subjects stood in this position while the researcher took a photograph. The process was repeated for a second photograph with the instructions: “March in place for a few steps, take a deep breath in and out, then assume your normal, relaxed posture.”

## MEASUREMENT OF CRANIOVERTEBRAL ANGLE

Craniovertebral angles were measured by two researchers blinded to the subject's identity using Image–J^®^ (Rasband, 1997). All the measurements were performed by two final year DPT students who were blind to each other's measurements and had completed the musculoskeletal part of the curriculum. Student researcher 1 (R1) measured both ideal posture and relaxed posture images. Student researcher 2 (R2) measured only relaxed posture images. Each image was measured three times on three different days and the mean was used for analysis.

## DATA ANALYSIS

Data analysis was performed using NCSS 8^®^ (LLC Kaysville, Utah, USA). The descriptive statistics of the sample were calculated. Normality of distribution was tested using the kurtosis and skewness of the frequency distribution of the CV angles. Measurements taken by R1 of the relaxed posture images were used to calculate intra-rater reliability. Measurements taken by R1 and R2 of the relaxed posture images were used to calculate inter-rater reliability. Inter-rater reliability was tested using an ICC model 2 and the mean of three image measurements [ICC (2,3)]. Intra-rater reliability was tested using an ICC model 3, also using the mean of three image measurements [ICC (3,3)]. Additionally, intra- and inter-rater reliability for asymptomatic and symptomatic subgroups were also assessed. Intraclass correlations and standard error of measurement (SEM) were manually calculated from the mean square values obtained from a repeated measure ANOVA. Intraclass correlations were interpreted based on the guidelines provided in Portney and Watkins (2009) Foundations of Clinical Research: Applications to Practice (3rd ed).

## RESULTS

**Figure 2:**
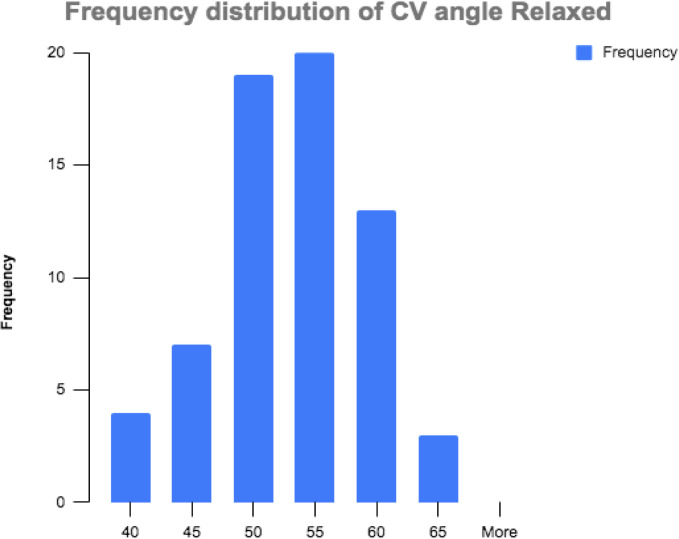
Relaxed Posture Histogram

**Figure 3:**
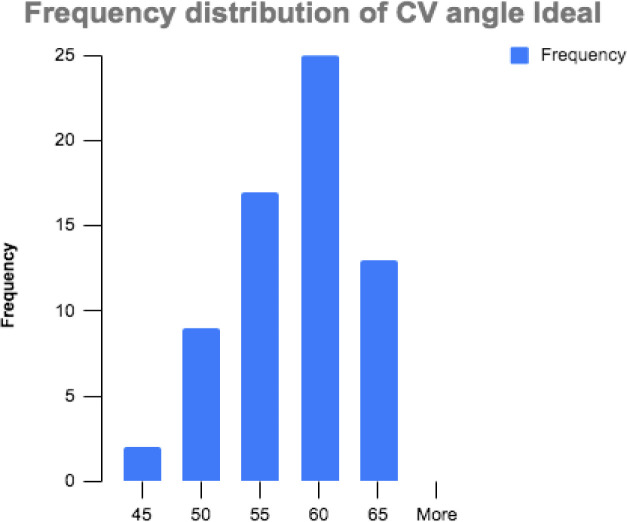
Ideal Posture Histogram

Demographic data is presented in Tables 1.1 and 1.2. The rater reliability and SEM values are presented in Table 2. Briefly, both the relaxed and ideal posture CV angles were normally distributed in our sample. The mean and standard deviation (SD) of relaxed posture was 50.7° ± 6.3° with kurtosis and skewness of 0.67 and −0.74 respectively (Figure 2). The mean and SD of ideal posture was 55.5° ± 5.4° with kurtosis and skewness of 0.1 and −0.54 respectively (Figure 3).

**Table 1.2:** Demographic Data

Other Variables	
Race	White 50
	Asian 7
	Latino 2
	Black 2
	Middle Eastern 1
	White/Latino 3
	White Asian 3
Gender	Female 56
	Male 10
Occupation	SPT 48
	DPT or MPT 18
Pain	Symptomatic 11
	Asymptomatic 55
Spinal Surgery	None 62
(Unspecified Location)	Surgical Intervention 4

**Table 2:** Reliability

	Relaxed Posture (ICC)	SEM
Inter-rater whole group (ICC 2,3)	0.88	1.87
Intra-rater whole group (ICC 3,3)	0.91	1.89
Intra-rater symptomatic (ICC 3,3)	0.99	0.72
Intra-rater asymptomatic (ICC 3,3)	0.99	0.59
Inter-rater symptomatic (ICC 2,3)	0.99	2.22
Inter-rater asymptomatic (ICC 2,3)	0.93	0.89

## DISCUSSION

The purpose of this study was to assess the rater reliability of CV angle measurement using a video-based telehealth platform. The results show that CV angles can be measured reliably by DPT students with limited clinical experience. This finding is important because of social distancing guidelines and increased utilization of telehealth platforms. Due to the COVID-19 pandemic, many clinicians and patients must interact in a virtual environment and most clinical measurements have only been tested for their psychometric properties for in-person clinical assessments (Exum et al., 2020; A. C. Lee, 2020). Testing the psychometric properties of clinical measurements in a virtual environment is crucial for accurate documentation and tracking patient progress during the COVID-19 pandemic. It is to be expected that with more widely available technology, and the expansion of telehealth into rural and low-income areas, telehealth will remain a key medium for health care delivery in the future (Pandemic Brings Telehealth to the Forefront, 2020).

The generalizability theory states that reliability of any measurement exists in relation to a specific set of testing conditions called facets (Portney & Watkins, 2009). The specific set of facets for testing CV angle differs between measurements in a clinical setting as opposed to a virtual setting. For example, the clinical setting provides greater control of the testing conditions. In contrast, in a virtual environment the therapist may be unable to control multiple factors including image quality, lighting, and accuracy of the patients' understanding of instructions, etc. It is not beyond reason to expect that the inherent differences between the clinical and virtual assessment environment will affect measurement reliability.

In this study a specific set of instructions were provided to all subjects for standardization of ideal and relaxed posture. For the ideal posture condition, subjects were told, “March in place for a few steps then assume your best posture.” For the relaxed posture condition, subjects were told “March in place for a few steps, take a deep breath in and out, then assume your normal, relaxed posture.” The reliability coefficients in this study for relaxed posture were 0.88 for inter-rater and 0.91 for intra-rater, which are comparable to the values reported in the literature (Table 3) (Akodu et al., 2018; Cureton, 1941; Kocur et al., 2019; Lau et al., 2010; Nam et al., 2013; Raine & Twomey, 1997; Salahzadeh et al., 2014; Subbarayalu, 2016). A plausible explanation for this might be that similarities that exist across clinical and virtual environments. For example, the method of measuring photometric CV angle is the same regardless of the setting the photograph is taken, whether virtual or in person (Cureton, 1941; Raine & Twomey, 1997; Salahzadeh et al., 2014).

**Table 3:** A Comparison of Reliability Values Reported in the Literature with the Current Study

Article (Author, Year Published)	N	Mean	Standard Deviation	Interrater ICC	Inter-rater ICC
Cureton, 1941	382	53.6	6.4	----	----
Raine, 1997	160	53.7	1.9	----	0.91
Lau, 2009	53	50.6	2.1	0.85-0.91	0.86-0.94
Nam, 2013	45	60.1	3.1	0.75	0.91
Salazahdeh, 2014	78	48.5	3.2	0.92	0.90
Subbarayalu, 2016	85*	47.5	2.5	0.76	0.87
Akodu, 2018	77	51.8	5.7	----	----
Kocur, 2019	50^	50.6	2.1	---	---
**Present Study, 2020**	66**	**50.6**	**6.2**	**0.88**	**0.91**

* 65 ' symptomatic and 20 = asymptomatic

** 11= symptomatic and 55 = asymptomatic

^ 25 = with FHP and 25 = with normal head posture

Another important facet of reliability is the accuracy of palpation of the landmarks used to measure the CV angle. Inaccuracy in identifying these landmarks can adversely affect reliability. Our sample consisted of DPT students and licensed clinical physical therapists. It is possible that their knowledge of anatomy and landmarks affected the reliability values favorably. Although the average CV angle values in this study were similar to those reported in the general population, it is possible that the reliability of CV angle measurements might be different in a sample taken from the general population. Specifically, with respect to CV angle measurements, clear instructions to identify landmarks or using a schematic diagram designed for individuals without anatomical knowledge might be helpful.

The photographs were taken after two different sets of instructions: (1) ideal posture as understood by the subject; and (2) relaxed posture. We found that there was a difference of 5.2 degrees between the two conditions. Our sample exclusively consisted of DPT students and PT clinicians. It is safe to assume that this subgroup is probably more aware of “good posture” than the general population. However, a difference of 5.2 degrees between the ideal and relaxed posture even in this subgroup raises questions about the effectiveness of patient education on ideal posture.

The relaxed posture CV angles were normally distributed with a mean of 50.6 degrees and standard deviation of 6.2 degrees. In the past, these mean values have been used to set a cutoff point for discriminating FHP from “normal posture.” Most of our sample was asymptomatic with only 11 symptomatic subjects. The mean CV angle of the symptomatic subjects was 50.5 degrees and SD 7.2 degrees compared to asymptomatic subjects with a mean of 50.5 degrees and SD of 5.9 degrees. Because of this overlap, we think that the future studies should explore the use of a different cutoff point for identifying forward head posture.

In addition to planned calculations, the intra-rater and inter-rater reliability for asymptomatic and symptomatic groups were calculated. The values are presented in Table 2. These numbers suggest CV angle is a reliable measurement over telehealth platforms as a measurement of forward head posture in populations with and without symptoms of cervical dysfunction.

The ICC value for symptomatic groups was higher than the ICC for asymptomatic groups. There are three plausible explanations for why this was the case. First, more symptomatic subjects enrolled in the study towards the end of the data collection period than earlier on. This could suggest that there was some practice effect on the raters' measurements. Second, the raters' measurements might have been more accurate when measuring symptomatic subjects versus asymptomatic subjects. The subgroup analysis was done post hoc and raters were not blinded to the condition of the subject. Third, breaking the sample into asymptomatic and symptomatic subgroups reduced the sample sizes. This is especially true for the symptomatic group, which only had 11 subjects. In addition, the within group variability for the symptomatic group was higher with a SD of 7.2 degrees compared to a SD of 6.0 degrees in the asymptomatic group. A larger difference between subjects' variance will increase the between subject mean squared value used to calculate ICC. This might have inflated the ICC values for the symptomatic group, and any generalizations beyond the study should be done with caution.

The findings of the present study should be interpreted with caution due to some limitations. The sample in this study consisted of only physical therapists. However, our aim was to assess the reliability of measuring CV angle and not a comparison of CV angles among different populations. Another limitation of this study was the inherent inconsistencies of telehealth platforms. Even though we tried our best to standardize the instructions given to the subjects, there was variability in lighting, camera setup, angle, subject background, height and placement of the camera, resolution of the photograph, visibility of the C7 marker, and the device used to take photographs. Despite these inconsistencies, the reliability coefficients were high suggesting that reliable measurement of CV angles could be performed despite variabilities that likely exist across most virtual environments.

## CONCLUSION

This study found acceptable levels of rater reliability for CV angle measurement over a video communication platform. While our measurements were not compared to in-person measurements of the same subjects, our means were similar to those found by previous researchers. These results suggest that CV angle can be utilized as a physical therapy telehealth assessment to provide object measurements as a potential basis for goal setting.
